# Prediabetes and Right Ventricular Function: What Should Be Done for Clarification?

**Published:** 2019-10

**Authors:** Tahereh Davarpasand

Dear Editor-in-Chief

The American Diabetes Association has opened a dysglycemic category termed “prediabetes”. This category includes subjects with fasting blood sugar levels of between 100 and 125 mg/dL or glycated hemoglobin A1c levels of between 5.7% and 6.4% or 2-hour post-glucose loads (75 g) of between 140 and 199 mg/dL.^1^ This category has been taken into consideration in cardiovascular disease research, including the cardiac imaging field. On the other hand, the right ventricular (RV) function has been a focal point in some investigations. 

In one study, a group of researchers compared 3 groups of euglycemic, prediabetic, and diabetic subjects according to the systolic and diastolic longitudinal deformations of the RV free wall obtained via 2D speckle-tracking echocardiography and the right ventricular ejection fraction (RVEF) measured with 3D echocardiography.^2^ The investigators found no statistically significant differences between the 3 groups regarding the RVEF, but the absolute values of systolic strain, the strain rate, and the early diastolic strain rate were lower in the prediabetic and diabetic subjects than in the euglycemic group. Additionally, the late diastolic strain rate was lower in the diabetic subjects than in the euglycemic subjects, and the deformation indices in the prediabetic subjects were not different from those among the diabetic patients. The study suffered from the exclusion of patients with coronary artery disease.

Elsewhere, another group of researchers included subjects with insignificant coronary artery stenosis documented by selective coronary angiography.^3^ The investigators compared the RV free-wall deformation indices in the aforementioned 3 glycemic state groups. Their adjusted analysis revealed that diabetes and prediabetes were the independent determinants of systolic strain, the strain rate, and the early diastolic strain rate. This study was stronger than the previous study given the exclusion of patients with significant coronary artery disease; however, microvascular coronary artery disease probably had a significant prevalence in the subjects of the study. 

The difference between the results of these 2 studies suggests 2 hypotheses that change from the euglycemic state to the dysglycemic state or a stage-by-stage aggravation in the RV function concomitant with the aggravation of the glycemic state may lead to RV dysfunction.^3^ To test these hypotheses, we propose cardiac magnetic resonance imaging (CMR) for the evaluation of the RV function. Although in a recent study, the RVEF as measured with CMR was not different between these 3 glycemic groups,^4^ we need deformation measurements in CMR so as to clarify the effects of the prediabetes state on the RV function.
